# 3.0-tesla magnetic resonance imaging in the assessment of postmenopausal osteoporosis: are technological advances capable of replacing bone densitometry?

**DOI:** 10.1590/0100-3984.2022.55.6e2-en

**Published:** 2022

**Authors:** Flavia Martins Costa

**Affiliations:** 1 Radiologist and MRI coordinator at Diagnósticos da América (DASA-RJ); Professor of Radiology at the Universidade Federal do Rio de Janeiro (UFRJ) School of Medicine, Rio de Janeiro, RJ, Brazil.

By 2050, approximately 22% of the world’s population is expected to over 60 years of age. The increase in life expectancy and consequent aging of the population have led to a higher prevalence of chronic non-contagious diseases, which are now considered to constitute a new “epidemic”^(1)^. One of the chronic non-contagious diseases that represents a risk to the health of the elderly population is osteoporosis^(1,2)^.

Osteoporosis is a metabolic bone disease that is prevalent in the elderly, predominantly in postmenopausal females; the resulting bone loss facilitates the occurrence of osteoporotic fractures, which have a great impact on quality of life, causing a significant loss of functionality, as well as increasing morbidity and mortality^(3)^. Given the multifactorial nature of the disease, prevention and early diagnosis are the greatest allies for healthy aging. Bone densitometry plays a decisive role in the screening and monitoring of individuals at risk for osteoporosis.

Since 1993, dual energy X-ray absorptiometry (DEXA) has been recommended by the World Health Organization as the method of choice for the quantification of bone mass, being used for the diagnosis and therapeutic follow-up of osteoporosis( 4). Since 2014, DEXA has been the diagnostic method of choice in Brazil because it is noninvasive, uses extremely low doses of radiation, provides an accurate assessment of fracture risk, and is the most suitable means available for evaluating individuals at risk of developing osteoporosis^(5,6,7,8)^. However, despite the fact that DEXA is a established method in the management of osteoporosis, a question arises^(9)^: Is there is a revolutionary high-tech examination that does not involve the use of ionizing radiation, is accessible, is replicable, and is capable of replacing densitometry? Trentadue et al.^(10)^ conducted advanced research using 3.0-tesla magnetic resonance imaging (MRI), including diffusion-weighted imaging (DWI) and apparent diffusion coefficient mapping, as a means of screening for and diagnosing osteoporosis in postmenopausal patients.

A 3.0-tesla MRI scanner employs the strongest magnetic field allowed in humans, with advanced technology, excellent multiplanar capacity capable of generating high-resolution images and high tissue contrast, thus enabling accurate diagnoses of numerous conditions involving the central nervous system, the cardiovascular system, the abdominal/pelvic organs, and the musculoskeletal system, especially the bone marrow^(11)^.

Although DWI is the method of choice for bone marrow assessment, there are numerous factors that influence the appearance of the bone marrow, including age, metabolic diseases, anemia, hematopoietic marrow reconversion, hematological disorders, and metastatic tumors^(11)^. Those factors are prevalent after the age of 60 and make it difficult to perform an isolated DWI assessment of the bony framework, which is composed of mineralized bone, thus preventing the accurate analysis of the reduction in bone mass density necessary for the diagnosis of osteopenia and osteoporosis.

We must recognize that MRI technology, including advanced techniques, is not superior to bone densitometry, which has proven to be more cost-effective for the early detection of osteoporosis, due to its low cost and lack of contraindications. In contrast, 3.0-tesla MRI not only is expensive and difficult to access but is also contraindicated in many situations, such as in patients with claustrophobia or with a pacemaker, as well as having limits regarding patient weight and abdominal circumference, and it still requires patient collaboration, which makes it difficult to replicate at scale.

Given that the aging of the population will become one of the most significant social transformations of the 21st century, with implications for various sectors of society, especially the health care sector, we need to contribute in the field of diagnosis effectively and accurately to promote healthy longevity^(12)^. It is therefore important to implement and operationalize health care for the elderly with preventive, treatment, and rehabilitation activities to maintain functional capacity and quality of life^(12,13,14)^.

Our mission is to identify high-tech diagnostic methods or methods that are capable of actively contributing to individual and collective actions, aiming at specific prevention, early diagnosis, and, consequently, appropriate treatment of the main health problems in the elderly population, including chronic non-contagious diseases^(15,16)^.
